# Response of Parasite Community Composition to Aquatic Pollution in Common Carp (*Cyprinus carpio* L.): A Semi-Experimental Study

**DOI:** 10.3390/ani13091464

**Published:** 2023-04-25

**Authors:** Markéta Pravdová, Jitka Kolářová, Kateřina Grabicová, Michal Janáč, Tomáš Randák, Markéta Ondračková

**Affiliations:** 1 Czech Academy of Sciences, Institute of Vertebrate Biology, Květná 8, 603 00 Brno, Czech Republicjanac@ivb.cz (M.J.); 2 Department of Botany and Zoology, Faculty of Science, Masaryk University, Kotlářská 2, 611 37 Brno, Czech Republic; 3 Faculty of Fisheries and Protection of Waters, South Bohemian Research Centre of Aquaculture and Biodiversity of Hydrocenoses, University of South Bohemia in České Budějovice, Zátiší 728/II, 389 25 Vodňany, Czech Republic; kolarova@frov.jcu.cz (J.K.); grabicova@frov.jcu.cz (K.G.); trandak@frov.jcu.cz (T.R.)

**Keywords:** condition, ectoparasites, endoparasites, environmental load, fish parasites, pharmaceuticals, sewage treatment plant

## Abstract

**Simple Summary:**

The impacts of pollutants on the aquatic environment have become an increasingly important subject of study over the past few decades. Pollutants, including pharmaceuticals, can have direct and/or indirect effects on biota, affecting individual trophic levels in the food chain, the composition of populations, or even the degree of parasitism, a secondary stressor to the host. In this study, we assessed uptake of pharmaceutical compounds in tissues of common carp (*Cyprinus carpio* L.) and parasite community response to the change in environmental conditions six months after relocation from a control to a treatment pond loaded by organic pollution from a sewage treatment plant outlet using partial cross-over experimental design. By comparing fish from control and treatment ponds, we observed higher pollutant uptake and the concentration of pharmacological compounds in fish tissues restocked to the treatment pond, along with changes in fish biometric parameters and parasite load. Fish from polluted environment exhibited decreased parasite diversity and higher ectoparasite abundance; however, the major differences were observed between families within taxonomic groups. Our results, therefore, highlight the need for more detailed taxonomic analyses in studies using parasites as potential environmental bioindicators.

**Abstract:**

The response of parasite communities to aquatic contamination has been shown to vary with both type of pollutant and parasite lifestyle. In this semi-experimental study, we examined uptake of pharmaceutical compounds in common carp (*Cyprinus carpio* L.) restocked from a control pond to a treatment pond fed with organic pollution from a sewage treatment plant and assessed changes in parasite community composition and fish biometric parameters. The parasite community of restocked fish changed over the six-month exposure period, and the composition of pharmaceutical compounds in the liver and brain was almost the same as that in fish living in the treatment pond their whole life. While fish size and weight were significantly higher in both treatment groups compared to the control, condition indices, including condition factor, hepatosomatic index, and splenosomatic index, were significantly higher in control fish. Parasite diversity and species richness decreased at the polluted site, alongside a significant increase in the abundance of a single parasite species, *Gyrodactylus sprostonae*. Oviparous monogeneans of the Dactylogyridae and Diplozoidae families and parasitic crustaceans responded to pollution with a significant decrease in abundance, the reduction in numbers most likely related to the sensitivity of their free-living stages to pollution.

## 1. Introduction

Freshwater ecosystems have long been subjected to serious threats from human activities, with pollution among the most significant [1,2,3]. Over recent years, there has been increasing concern over the unintentional presence of pharmaceuticals and personal care products (PPCPs) in different compartments of the aquatic environment (e.g., water, sediments, and biota) at concentrations capable of causing detrimental effects to aquatic organisms [4]. Indeed, PPCPs have now been detected worldwide in treated sewage, rivers and streams, seawater, groundwater, and drinking water [5]. In part, this is because present conventional systems of wastewater treatment are not designed to fully remove these contaminants [6]. Though a wide range of pharmaceutical classes are used in human and veterinary medicine, only a few are considered of environmental importance due to their consumption volumes, toxicity, and/or persistence in the environment [7,8]. Despite this, little is known about the ecotoxicological effects of PPCPs on non-target aquatic organisms exposed to such wastewater residues over their life [7], or their natural interactions [8].

The majority of fish populations are exposed to a wide variety of anthropogenically sourced chemical compounds, including PPCPs. Though these chemicals are often found at concentrations not directly toxic to fish, exposure at sublethal concentrations may still induce harmful effects [9]. Aquatic habitats under chronic exposure to pollutants often suffer reduced species richness and a loss of community integrity, which may subsequently impact the whole ecosystem [10]. Further, when subjected to many pollutants, other common natural stressors, such as parasites and pathogens, may have an additional effect on fish host physiology and population structure [8]. The combined effects of multiple stressors, i.e., parasites and pollution, may then subsequently reduce either fish host resistance or tolerance to infection [11]. On the other hand, aquatic contaminants may also affect the parasites. As the parasites are, for part of or their whole life, in direct contact with their environment, and thus the toxic substances, their vitality may decrease or mortality increase, with subsequent impacts on parasite community composition and diversity [12]. Despite an increasing number of recent field studies, our understanding of the synergistic effects of pollution and parasites on fish host populations remains limited.

Fish parasite abundance, community composition, and structure may be affected by a range of factors, including host ecology and physiology, environmental factors, and anthropogenic stressors [13,14]. Pollution, and other anthropogenic disturbances to the aquatic environment, may affect a parasite community directly by acting on free-living parasite stages [15,16] or on ectoparasites [17], or indirectly by acting on intermediate or definitive host populations [13,18]. This wide variability in the effects of aquatic contaminants on parasites can cause alterations at both organism and population levels [19]. For example, increases in parasite levels in the affected environment may reflect stress-related immune suppression and reduced resistance in the host [20]. Alternatively, an increase in intermediate host abundance due to improved living conditions, e.g., following eutrophication, may favor parasite transmission [13], while decreases in parasitism may result from direct toxicity to either parasites [15] or their intermediate hosts, leading to a decrease in abundance [13].

As early as the 1980s, Möller [18] postulated that the composition of ectoparasitic fauna in aquatic organisms might be a useful and quickly reacting indicator for the effects of certain pollution conditions in freshwater ecosystems. A later summary by Blanar et al. [21] confirmed this, with the authors showing that environmental pollution generally has a stronger, mostly negative, effect on directly exposed parasites, i.e., free-living stages and ectoparasites, when compared with endoparasites. Moreover, monoxenous parasites, i.e., parasites with direct life cycles, tend to show higher susceptibility to a larger variety of environmental stressors [21]. Most such studies, however, have been focused on eutrophication and heavy metal pollution [12,22,23], while the effect of pharmaceutical pollutants on host–parasite interactions remains less explored [8,24,25].

In the present study, a partial cross-over experimental design was used to assess uptake of pharmaceutical compounds in tissues of common carp (*Cyprinus carpio* L.) six months after relocation from a control pond to a treatment pond, and to assess parasite community response to the change in environmental conditions. As Blanar et al. [14] pointed out, comparisons of localities in ecotoxicological studies usually consider polluted (treatment) localities without any deep specification of the pollutants involved. In our study, we determined pharmaceutical pollutants as high concentrations of PPCPs have recently been detected in both fish and water samples from the treatment pond [26,27,28], while heavy metal concentrations at the same locality are generally low [29]. Although the fish parasite infection was not evaluated prior the experiment, previous studies in the study area (i.e., pond system in Vodnany region) indicate that parasite communities of common carp share similar parasite species with dominance of monogenean and cestode parasites [25,30], but they differ in parasite prevalence and abundance, reflecting seasonal changes [25,30] and water quality [25]. By relocation of experimental fish in both directions, similar initial pool of parasites was achieved at both sites. The experimental design of our study therefore allowed us to assess how parasites relocated to environmental conditions affected by a high concentration of PPCPs cope with such a change. We hypothesized that parasites sensitive to pollution will limit their reproduction during the main growing season, leading to low abundance at the end of the experiment. Parasites that can take advantage of their host being weakened by other stressors were then expected to increase in their abundance, particularly in restocked fish, as original fish were predicted to be longer adapted to the polluted environment.

## 2. Material and Methods

### 2.1. Study Area and Experimental Design

For this study, common carp were collected from two experimental ponds with different pollution levels. The treatment locality (Cezarka pond; 49.1466617 N, 14.1915231 E) is a treated wastewater pond (2.6 ha, average depth 1–2 m) located below the sewage treatment plant (STP) for the town of Vodnany (Czech Republic). The STP runs a standard treatment process, including mechanical filtration, sedimentation, biological treatment, sludge concentration, and secondary settlement in tanks. The treated effluent is then fed directly into Cezarka pond, which provides wastewater stabilization as a tertiary treatment process prior to introduction into a fish production pond. STP effluent (except for precipitation) is the sole source of water for the pond. In recent years, studies have recorded high concentrations of PPCPs in both fish and water samples from the pond [27,28,31]. The control pond (49.1577281 N, 14.1623011 E; 0.12 h, average depth 1 m), located near the University of South Bohemia’s Faculty of Fisheries and Protection of Waters in Vodnany, was selected as a locality with no significant pollution [32], being fed by water from the adjacent River Blanice, which has low background pollution concentrations typical of a regional river. As the two ponds are approximately 2 km apart, they are both subject to comparable climatic conditions.

Both ponds were stocked with common carp of the same genetic origin that were allowed to feed on natural food only, i.e., there was no supplemental feeding with grain. For the first year of life, the fish were reared in the Cezarka treatment pond and control pond under standard rearing conditions. In April 2018, both ponds were harvested and one year-old fish of similar size were selected from each pond and group-marked. Two groups were then created from the fish from each pond, resulting in four groups for further monitoring, i.e., T-T (original fish from treatment Cezarka pond, returned to Cezarka); T-C (fish originally from Cezarka treatment, restocked to control pond), C-C (original fish from control pond, returned to control pond), and C-T (fish originally from control pond, restocked to Cezarka treatment pond). The ratio between stocked/restocked fish for the two groups in both ponds was approximately 1:1, with final density in both ponds being 0.14 fish × m^−2^ (see [32] for further details). The fish were kept in particular ponds for a period of six months, corresponding to the length of growing season in this type of habitat. After six months (October 2018), fish were collected from both sites using seine and gill nets until 20 fish from group T-T, 20 from group C-T (both from the Cezarka treatment pond), and 20 from group C-C (control pond) had been collected. Unfortunately, due to technical problems, fish from group T-C (originating from Cezarka, restocked to the control pond) could not be used for further analysis; thus, this study only represents a partial cross-experiment.

### 2.2. Fish Processing and Chemical Analysis

The fish from each treatment group (T-T, C-T, C-C; *n* = 20 per group) were humanely dispatched prior to dissection for subsequent parasitological and chemical analysis and the standard length (SL, mm), total length (TL, mm), and total weight (Wt, g) recorded. The fish were then eviscerated and the eviscerated body weight (W_E_, g) taken, after which the weight of the liver (W[liver], mg), spleen (W[spleen], mg) and gonads (W[gonads], mg) were recorded. Four fish body condition indices were then calculated for each fish, i.e., condition factor (K) = W_E_ × 10^5^/SL^3^; hepatosomatic index (HSI) = W[liver] × 10^2^/ W_E_; splenosomatic index (SSI) = W[spleen] × 10^3^ W_E_; and gonadosomatic index (GSI) = W[gonads] 10^2^/W_E_. For some fish, it was not possible to retrieve the gonads, and thus confirm the sex; in which case, the fish was not included in any further analysis. W_E_ was used rather than Wt for condition factor analysis to eliminate errors caused by different levels of stomach fullness [33].

Liver and brain tissue samples (0.5 g) were taken from each fish during dissection, and the concentration of 69 pharmaceutical compounds was determined according to validated methodologies [34]. Briefly, isotopically labeled internal standards, an extraction solvent, and a homogenizing ball were added to the pre-weighed tissue, after which it was homogenized and centrifuged. The supernatant was then filtered (regenerated cellulose, 0.45 µm pores), frozen at −20 °C for 24 h, then defrosted and an aliquot analyzed via liquid chromatography using a high-resolution mass spectrometer (Thermo Fisher Scientific, Waltham, MA, USA). For further analysis, all pharmaceuticals detected were sorted into medicinal classes according to their clinical effects (Supplementary Tables S1 and S2).

### 2.3. Parasite Collection and Identification

All fish were examined for the presence of parasites under a binocular microscope using standard protocols. Protozoan parasites living on gills and fins were examined under light microscopy. Metazoan parasites collected were preserved in glycerin ammonium-picrate mixture (monogeneans), 4% formaldehyde (cestodes, crustacean, hirudineans, glochidia), or 70% ethanol (nematodes). Cestodes were stained using iron acetic carmine, dehydrated in ethanol of increasing concentration and then mounted in Canada balsam as permanent slides [35], while nematodes were mounted in glycerol as temporary slides for light microscopy. Larval parasites, impossible to identify using standard morphological methods (e.g., larval trematodes), were preserved in 96% ethanol and identified using molecular methods following Georgieva et al. [36]. All parasites were identified to species level or to the lowest possible taxa following the respective keys [37,38,39] under an Olympus BX51 light microscope equipped with phase contrast and Stream Motion v.1.9.2 digital image analysis software (Olympus, Tokyo, Japan). Levels of parasite infection were expressed as prevalence, calculated as the proportion of parasitized fish from all fish in each group, mean abundance was expressed as the mean number of parasites in all hosts in the sample and intensity of infection was calculated as the mean number of parasites in infected hosts [40]. All infection parameters were calculated for individual parasite species. Parasite diversity was measured using the Species richness, Shannon–Wiener, Dominance, and Equitability indices for all fish in each group [41], with diversity index values calculated using PAST software [42]. The same software was also used to compare index values between parasite communities using permutation tests generating 1000 random matrices with two samples, the *p*-value being computed as the proportion of randomly permutated matrix combinations resulting in index difference higher or equal to the difference observed.

### 2.4. Data Analysis

Inter-group differences in composition of both parasite community and pharmaceuticals were visualized using non-metric multidimensional scaling (NMDS) and tested using permutational multiple analysis of variance (PERMANOVA). Parasite data were fourth-root transformed and Bray–Curtis distances were used to describe the sample distances. Pharmaceutical data were log (x + 1) transformed, scaled, and centered (to a mean of 0 and SD of 1) and Euclidean distances were used to describe sample distances.

Inter-group differences in the four parasite assemblage characteristics (i.e., species richness, abundance, abundance of *Gyrodactylus* spp., and abundance of *Dactylogyrus* spp.) were tested using generalized linear models (GLM; Poisson distribution for richness, negative binomial for abundance), with fish SL as a covariate. Owing to their low abundance, other parasite species were not compared. The Tukey HSD approach was used to control for type II errors in multiple post-hoc pairwise comparisons in all models (using the glht and mcp functions from the *multcomp* package; [43]). Inter-group differences in pharmacological load and fish biometric parameters were tested using analysis of variance (ANOVA) on log-transformed data, with Tukey HSD post-hoc pairwise comparisons.

Potential associations between parasites, pharmaceuticals, and fish biometric parameters were examined using co-inertia analysis (COIA, [44,45]), testing whether the variability in parasite assemblage structure, pharmacological load, and biometric parameters were pair-wise correlated to each other. For each fish group (C-C, C-T, T-T), separate COIA analyses were conducted. The first step of COIA involved separate principle component analyses (PCA) of three matrices: parasite abundance, concentration of pharmaceuticals (the same datasets that were used for inter-groups comparisons of parasite assemblage and composition of pharmaceuticals), and fish biometric parameters. In a second step, each pair of PCA ordinations was combined in COIA to explore the co-structure between them. The assessment of a possible link between the two tables was obtained by performing pairwise Monte Carlo permutation tests on the value of the RV coefficient (expressing the amount of correlation between matrices) using 999 random permutations. All statistical analyses were undertaken using R v.4.1.1 [46].

## 3. Results

### 3.1. Parasite Community

A total of 18 parasite taxa were recorded in the three groups of common carp. Of these, 16 were identified in the C-C group, 10 in the T-T group, and 10 in the C-T group (Table 1), with significantly higher species richness exhibited in control C-C compared with the treatment T-T site (permutation test, *p* = 0.001; Table 2). The parasite community mainly consisted of ectoparasites, with the dominance of monogeneans (Gyrodactylidae, Dactylogyridae, Diplozoidae) and crustacean (Ergasilidae, Argulidae) species. *Gyrodactylus sprostonae* was the most abundant and prevalent parasite in both the T-T and C-T groups from the Cezarka treatment pond. Dactylogyridae (*Dactylogyrus falciformis*, *D. achmerowi*, *D. extensus*, and *D. molnari*), along with *Eudiplozoon nipponicum*, dominated in fish from the control C-C group. In addition, a high prevalence of parasitic crustaceans (*Ergasilus sieboldi*, *Argulus coregoni*, *A. foliaceus*, *A. japonicus*) was also recorded in fish from the C-C group. Endoparasitic species, represented by one larval trematode and three cestode species, occurred relatively rarely (Table 1).

There were clear differences in parasite diversity between fish from the C-C group and those from the T-T and C-T treatment groups, with the C-C control having significantly higher Shannon–Wiener diversity (all *p* < 0.001) and Equitability indices (all *p* < 0.001) and a significantly lower Dominance index (all *p* < 0.001, Table 2).

**Figure 1:**
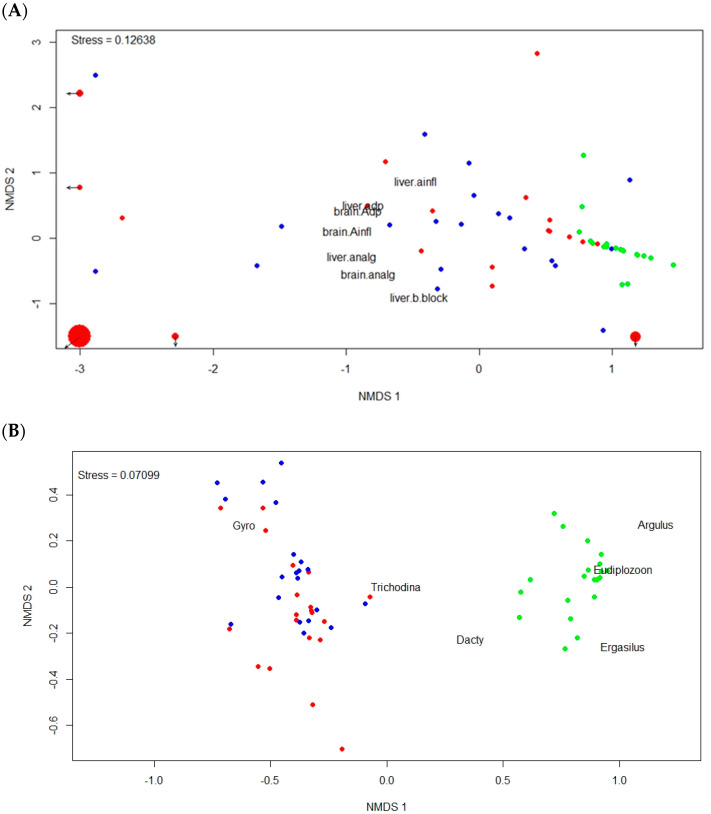
(**A**) Differences in parasite community composition, and (**B**) composition and concentration of pharmaceutical compounds summed for particular medicinal classes, i.e., antidepressants (ADP), analgesics, anti-inflammatory drugs (AINFL), and beta-blockers, observed in fish from T-T (persistent at treatment site; red marks), C-C (persistent at control site; green marks), and C-T (restocked from control to treatment site; blue marks) groups visualized on a non-metric multidimensional scaling (NMDS) graph. Outliers are shown as larger points (size corresponds to distance from the depicted area, arrows mark the direction).

**Figure 2:**
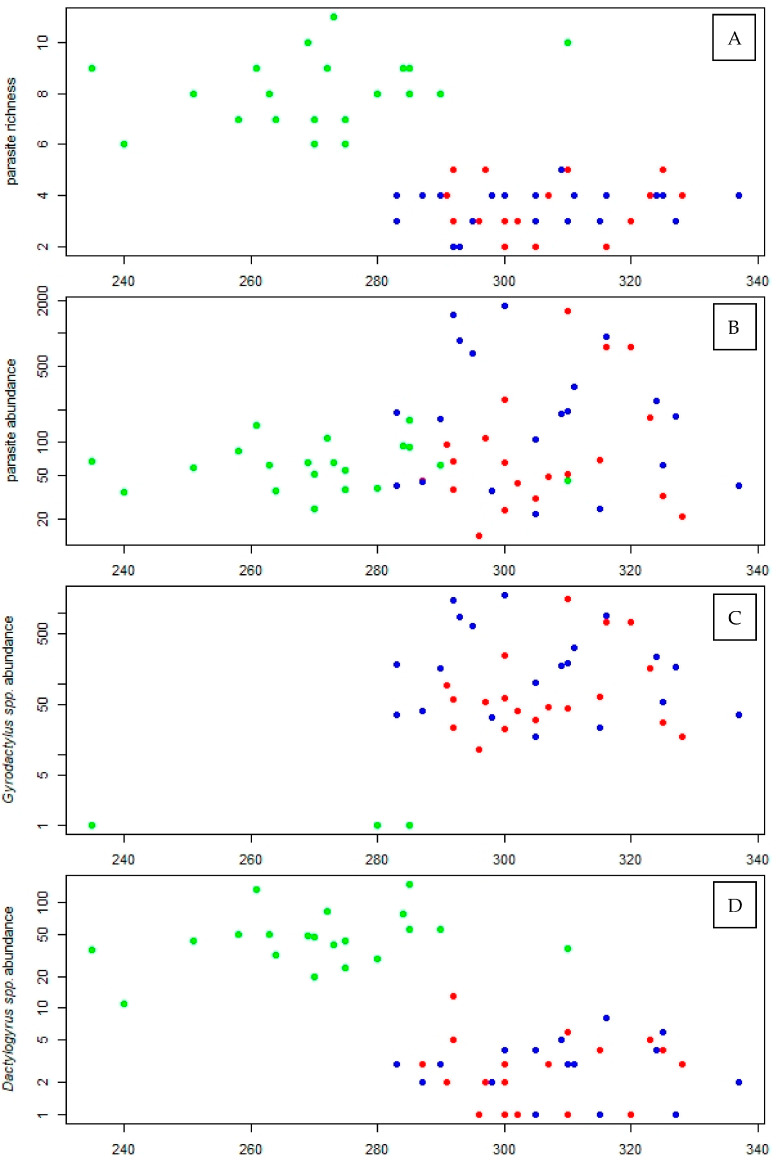
(**A**) Parasite species richness, (**B**) abundance of all parasites, (**C**) abundance of *Dactylogyrus* spp., and (**D**) abundance of *Gyrodactylus sprostonae* in fish from C-C (persistent at control site; green marks), T-T (persistent at treatment site; red marks), and C-T (restocked from control to treatment site; blue marks) groups, with standard length (SL) as the covariate.

Fish from the C-C group also had a significantly different parasite community composition compared to both the C-T and T-T groups (PERMANOVA, both *p* < 0.001, Table 3), with no significant differences between C-T and T-T fish (PERMANOVA, *p* = 0.058, Figure 1A). There were also significant differences in all four univariate parasite assemblage characteristics (GLM, all df = 2,56, all *p* < 0.001). While there was no difference in parasite species richness between T-T and C-T (post-hoc comparison, *p* = 1.000), both groups were significantly less rich compared to C-C (post-hoc comparisons, both *p* < 0.001; Figure 2A). Parasite abundance in C-T was significantly higher than that in C-C (post-hoc comparison, *p* = 0.002), while there was no significant difference in abundance between T-T and both C-C and C-T (post-hoc comparisons, *p* = 0.109 and 0.143; Figure 2B). Abundance of *G. sprostonae* in C-C was almost negligible, and significantly lower than that in either T-T or T-C (post-hoc comparisons, both *p* < 0.001), with no significant difference between the latter two (post-hoc comparison, *p* = 0.238; Figure 2C). In contrast, *Dactylogyrus* spp. abundance in C-C was significantly higher than that in both C-T and T-T (post-hoc comparisons, both *p* < 0.001), which again, were not significantly different from each other (post-hoc comparison, *p* = 0.801; Figure 2D).

### 3.2. Fish Biometric Parameters

**Figure 3:**
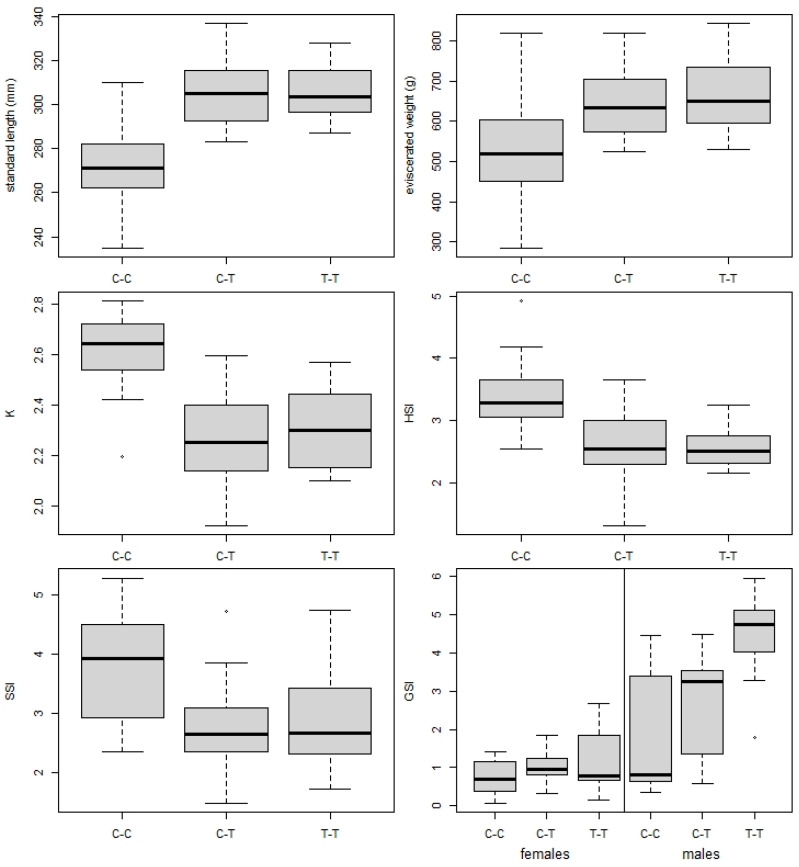
Comparison of biometric parameters between T-T (persistent at treatment site), C-C (persistent at control site), and C-T (restocked from control to treatment site) fish groups, showing standard length (SL), eviscerated body weight, condition factor (K), hepatosomatic index (HSI), splenosomatic index (SSI), and gonadosomatic index (GSI) for females and males. Horizontal line = median, box = interquartile range, whiskers = non-outlier range, points = outliers.

Fish SL and W_E_ were significantly lower in fish from the control C-C group compared with both the C-T and T-T treatment groups (ANOVA, F_2,57_ = 36.7, *p* < 0.001 and F_2,57_ = 11.9, *p* < 0.001, respectively; all Tukey post-hoc comparison tests *p* < 0.001). K, HSI, and SSI differed significantly between groups (ANOVA, F_2,57_ = 26.9, *p* < 0.001; F_2,57_ = 16.3, *p* < 0.001; F_2,57_ = 9.7, *p* < 0.001, respectively), with higher values observed in C-C compared to C-T and T-T (post-hoc comparisons, all *p* < 0.002; Figure 3). No significant differences were observed between C-T and T-T for all parameters mentioned above. For GSI, while no significant differences were found between groups for females (ANOVA, F_2,27_ = 1.3, *p* = 0.299), male fish from T-T showed higher GSI values than those from C-C (ANOVA, F_2,25_ = 6.4, *p* = 0.006, post-hoc comparison *p* = 0.004), with no difference between C-T and the other two groups (post-hoc comparisons, *p* = 0.237 and 0.133; Table 4).

### 3.3. Presence of Pharmaceuticals in Fish Tissues

**Figure 4:**
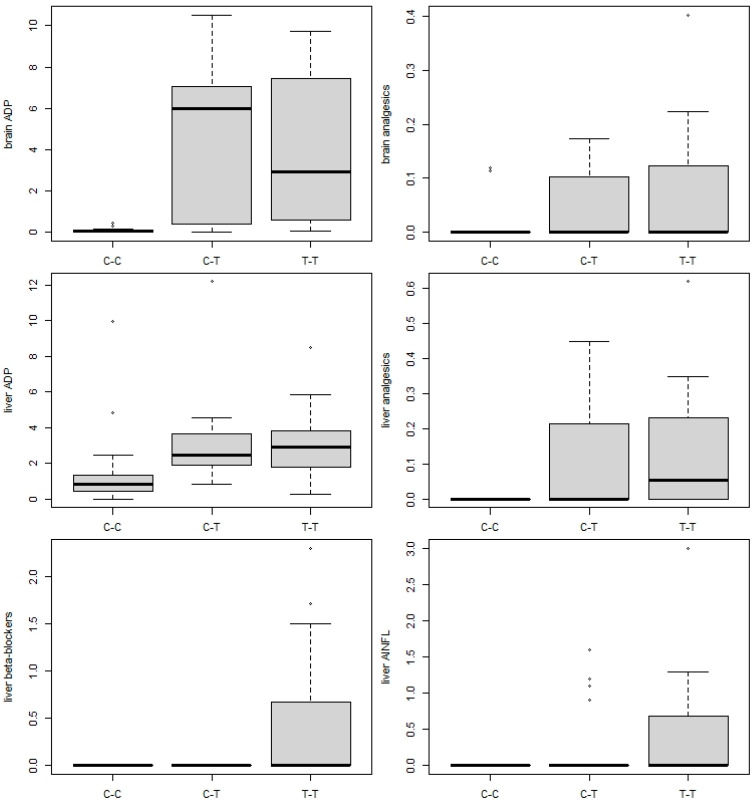
Comparison of mean concentrations of pharmaceutical classes (antidepressants (ADP), anti-inflammatory drugs (AINFL), beta-blockers, and analgesics) in the brain and/or liver for the T-T (persistent at treatment site), C-C (persistent at control site), and C-T (restocked from control to treatment site) fish groups. Horizontal line = median, box = interquartile range, whiskers = non-outlier range, points = outliers.

Of the 69 pharmaceuticals and/or metabolites determined in liver and brain tissue, 15 compounds were detected in at least one tissue type. Antidepressants and beta-blockers were the most frequently registered (eight and three compounds, respectively), with analgesics, anti-inflammatory drugs, antiepileptics, and CNS stimulants represented by a single compound each. In brain tissue, six, ten, and nine pharmaceutical compounds were found in the C-C, C-T and T-T groups, respectively, while four, nine, and eleven compounds, respectively, were found in the liver (see Supplementary Table S1). Fish in the C-C group had a significantly different composition of pharmaceutical loading compared to C-T and T-T fish (PERMANOVA, all *p* < 0.001; Table 5), with no significant differences between C-T and T-T fish (PERMANOVA, *p* = 0.179; Figure 1B). Antidepressants were found in significantly higher concentrations in both brain and liver tissue, and analgesics in liver tissue, in fish from the C-T and T-T groups compared with the C-C control (ANOVA, F_2,57_ = 17.89, *p* < 0.001, F_2,57_= 9.31, *p* = 0.001, and F_2,57_ = 6.54, *p* = 0.002; post-hoc comparisons all *p* < 0.005; Figure 4), with no significant difference between the C-T and T-T groups (post-hoc comparisons, *p* = 0.884, 0.997, and 0.942). Beta-blocker concentrations were significantly higher in fish from the T-T group than the C-C and C-T groups (ANOVA, F_2,57_ = 8.59, *p* < 0.001; post-hoc comparisons *p* = 0.002 and *p* = 0.002), while anti-inflammatory drug concentrations were significantly higher in T-T than C-C (ANOVA, F_2,57_ = 3.19, *p* = 0.049; post-hoc comparison *p* = 0.042), with no difference between the C-T and T-T and C-C groups (post-hoc comparisons, *p* = 0.232 and 0.688, respectively).

### 3.4. Association between Parasite Infection and Pharmaceutical Load

Significant covariance between parasite assemblage structure and composition of pharmaceutical load in fish tissues were found in both T-T and C-T fish but not in C-C fish (Table 6). No such covariance was observed between fish biometric parameters and either parasite assemblage structure or pharmaceutical load in all fish groups (Table 6).

## 4. Discussion

In this study, we addressed the effects of aquatic pollution on common carp by assessing the uptake of pharmaceutical compounds and their effect on biometric parameters, parasite community composition, and parasite abundance, using a partial cross-over semi-experimental approach. While changes in parasite community composition, diversity, and species richness have previously been used as an indicator of environmental impact [47], Marcogliese et al. [48] suggested that parasite communities may not be sensitive enough to detect the effects of low to moderate pollution, or that the effects may be overshadowed by those of natural environmental variation. In our study, the less polluted control site was characterized by higher parasite diversity and equitability and lower dominance. This corresponds to findings reported from a range of other freshwater ecosystems, e.g., [47,49,50,51], and supports the indication value of parasite communities in partially controlled natural studies. The parasite community of carp restocked into the treatment pond (C-T) from the control pond (C-C) adapted quickly to the new environmental conditions and, after six months exposure, matched the composition of those carp in the treatment pond. This change was manifested by a dramatic decrease in dactylogyrid and diplozoid monogeneans and ergasilid crustaceans and the disappearance of *Argulus* spp., alongside an increase in the abundance of gyrodactylid parasites. Though a detailed analysis of endoparasites could not be performed due to the generally low levels of infection, our data clearly show differences in parasite community composition between the control and treatment sites (Figure 1). Thus, unsuitable environmental conditions in Cezarka pond resulted in a decrease in parasites species richness and a decrease in the abundance of viviparous monogeneans and parasitic crustaceans, possibly due to the direct effect of toxic substances on free-living parasitic stages [52]. On the other hand, massive gyrodactylid infection levels in the treatment pond resulted in a significant decrease in parasite diversity and equitability, alongside high dominance indices.

Proliferation of viviparous gyrodactylid parasites tends to be attributed to a reduction in host resistance under certain pollution conditions [18]. Moreover, polluted environments are assumed to negatively affect fish gills and damage the skin’s protective barrier, facilitating access to infection [53]. As a result, weakened individuals are more likely to be infected by parasites, particularly on the host’s surface [54,55]. Alternatively, contaminant exposure has been shown to result in hosts producing excess mucous, which gyrodactylids feed on [56,57], possibly explaining the high gyrodactylid numbers at polluted sites. Thus, gyrodactylids may prosper in such polluted environments as their reproductive strategy allows them to reproduce rapidly, resulting in large-scale invasion of hosts [58]. These findings were supported by our own study, where hundreds of *G. sprostonae* were found on the fins and gills of a single common carp in both the C-T and T-T treatment groups, while only a few specimens infected control (C-C) fish. Overall gyrodactylid abundance was almost two-times higher in fish restocked to Cezarka pond (though the difference was not significant), indicating that stress associated with relocation to a polluted site may contribute to such high infection intensities. As summarized in Gilbert and Avenant-Oldewage [19], similarly high gyrodactylid abundance levels have been found in fish exposed to eutrophication [22] and sediments contaminated with polynuclear aromatic hydrocarbons (PAHs) and PCBs [59] or pharmaceutical compounds released from sewage treatment plant effluent [24]. Surprisingly, opposing results were observed in three-year-old carp in our previous study [25]; however, in this case, the absence of gyrodactylids in carp from the Cezarka treatment pond was explained by the extremely high condition status of the fish due to excess food availability [31], which helped the fish cope better with parasite infection [25]. In the present study, carp in the two treatment groups had much lower condition factors as a result of decreased natural food availability in autumn [31].

Unlike gyrodactylids, oviparous monogeneans, including dactylogyrids and diplozoids, and parasitic crustaceans, which were all abundant at the control site (C-C), were significantly reduced in fish from both treatment groups (T-T and C-T). Carp from the control site, which were naturally infected with a rich and abundant dactylogyrid community, lost most of their parasites after being restocked to the Cezarka treatment pond, retaining just 4% of dactylogyrids originally found at the control site. While almost all control fish were infected with *E. nipponicum*, for example, only one parasite was found in each of the treatment groups, suggesting that the free-living larval stage, the oncomiracidium, which actively searches for hosts after hatching in water, may be highly sensitive to environmental stress [15], with a resultant drop in numbers in polluted environments. A similar explanation may also be applied for the absence of *Argulus branchiuran* and the decrease in abundance of the ergasilid copepod *E. sieboldi* at the Cezarka treatment pond. A similar reduction in parasite load has been observed for oviparous monogeneans (*Cichlidogyrus* spp.) infecting Mozambique tilapia (*Oreochromis mossambicus*) in African reservoirs, where parasite abundance and species richness decreased dramatically with increased levels of contamination [60]. Likewise, a decrease in the diversity and abundance of *Dactylogyrus* spp. was also found in chub (*Squalius cephalus*) at downstream sites along an increasing pollution gradient in the River Bilina, Czech Republic [50]. Moreover, Gilbert and Avenant-Oldewage [17] found that the diplozoid *Paradiplozoon ichthyoxanthon* had disappeared from a polluted site in South Africa within 14 years of the water quality decreasing, despite the plentiful presence of its fish host, while changes in parasite prevalence in the control lake over the same period clearly reflected the parasite’s natural seasonal variance. It should be noted, however, that the response of dactylogyrid parasites to pollution varies between studies, with data analyzed from 14 published studies showing an equal number of positive and negative responses of dactylogyrids to contaminants (see [19]). While eutrophication has been reported as promoting the abundance of dactylogyrid and diplozoid monogeneans [22,61], we observed a reduction in infection in our study, possibly related to the composition of pharmaceutical compounds in the Cezarka treatment pond. For example, while antibiotics and antihelminthics were not detected in fish at significant concentrations during the study, these compounds are regularly present in the water [25,31] and may possibly have had a negative effect on the survival of free-living parasite stages.

Our data showed that fish restocked from the control (C-C) to the treatment pond (C-T) increased their growth rate, resulting in a significant increase in SL and Wt over six months that matched the values of fish living in the treatment pond their whole life (T-T group), while condition indices in the treatment group were significantly lower than those in control. However, no covariance was observed between fish biometric parameters and pharmaceutical load. In a previous study at the same site [31], extreme growth rates of juvenile carp in the Cezarka treatment pond led to a high total fish biomass within the first three months. The resultant high feeding pressure caused a significant reduction in the availability of natural food, leading to a dramatic decrease in K and his over the following three months due to high competition for food, though SL and Wt remained higher in the Cezarka pond [31], as in our own results. It is also likely that the massive gyrodactylid infection observed contributed to the decrease in carp condition parameters in both treatment groups.

As pharmaceuticals usually form complex mixtures with additive, synergistic, or antagonistic impacts under natural conditions [62], separation of impacts from individual pharmaceuticals is at the least complicated, if not impossible [63]. Variability in pharmacological load at treatment site was significantly associated with that in parasite assemblage structure in both C-T and T-T treatment groups, while no such association was found in C-C group, supporting our prediction of parasites response to organic pollution. Nevertheless, no significant differences in parasite abundance and species composition, as well as pharmaceutical load between C-T and T-T groups suggest that relocated fish quickly adapted to the new conditions, showing similar vulnerability to parasites as T-T fish. High pharmacological load in fish tissues have previously been associated with overall parasite abundance of brown trout exposed to STP effluent in a small river [24], suggesting that exposure to pollutants impairs the host’s immune system, resulting in a higher susceptibility to parasite infection [13,20,64]. Over the six-month period of exposure in this study, carp restocked to the Cezarka pond mainly acquired antidepressants and their metabolites (clomipramine, sertraline and its metabolite norsertraline, citalopram and its metabolite N-desmetylcitalopram), anti-inflammatory drugs (diclofenac), and analgesics (tramadol), with the antidepressants and analgesics reaching almost the same concentrations as fish living in the pond their whole life (T-T group; Figure 3), reflecting their relatively rapid accumulation in fish tissue, particularly the brain [65]. In the study by Pravdová et al. [24], the high concentrations of antidepressants in brown trout resulted in a significant increase in gyrodactylid parasites. Accordingly, our results showed massive gyrodactylid infection in common carp exposed to pharmacological pollution where antidepressants were among the most concentrated compounds (Supplementary Table S1). Recent studies examining the effects of neuroactive pharmaceuticals on fish behavior [66,67] have suggested that ecological endpoints (e.g., behavior) are more sensitive to pharmaceuticals than the more commonly used toxicological endpoints [68]. This is because such compounds are specifically designed to affect mood or nerve function and reduce stress, with possible consequences for feeding, predator avoidance, or schooling behavior [6]. Transmission of gyrodactylids from one host to another occurs through direct contact [58]. Increased gyrodactylid infection at the treatment site may, therefore, be also associated with behavioral changes potentially induced by neuroactive compounds [69,70], in addition to the negative impacts of contaminants on the immune response of the host fish enhancing the infection rate [13,20,71].

## 5. Conclusions

This semi-experimental study increases our knowledge of how fish and their parasites respond to organic pollution in aquatic habitats. By comparing fish from control and treatment ponds, it was possible to observe differences in both pollutant uptake and the concentration of pharmacological compounds in fish tissues, along with changes in fish biometric parameters and parasite load. Though our data confirmed higher ectoparasite abundance and a slightly lower endoparasite abundance in polluted environments, which agrees with general trends elsewhere [21], such trends are not universal, and major differences were observed between families within taxonomic groups, especially in ectoparasites. Monogeneans are often considered useful environmental bioindicators [12,19,59]; our data, however, show that their bioindicative abilities can vary widely among families. Our results, therefore, highlight the need for more detailed taxonomic analyses, at least to the levels of family or genus, in the parasites evaluated for studies using parasites as pollution indicators.

The experimental design of this study did not allow us to study consecutive development of parasite community in fish translocated to polluted environment, as all fish were dissected after six months growing season. In future studies, assessment of parasite communities in different phases of fish host exposure to organic pollution would help to clarify the differences between parasite groups in their response to inappropriate environmental conditions.

## Figures and Tables

**Table 1:** Prevalence (*p*, in %), mean abundance (A), and range (min–max) in the intensity of infection for parasites collected on common carp from the three test groups, i.e., T-T (persistent at Cezarka treatment pond), C-C (persistent at control site), and C-T (restocked from control to treatment site for six months).

	T-T			C-C			C-T		
Parasite Taxa	*p* (%)	A	Range	*p* (%)	A	Range	*p* (%)	A	Range
Ciliophora									
*Ichthiophthirius multifiliis*				15	0.20	(1–2)			
*Trichodina* spp.	80			100			95		
Monogenea									
*Gyrodactylus sprostonae*	100	207.75	(12–1594)	15	0.15	(1)	100	374.95	(18–1778)
*Dactylogyrus falciformis*	10	0.15	(1–2)	100	34.45	(2–105)	15	0.15	(1)
*Dactylogyrus achmerowi*	75	1.60	(1–8)	95	10.05	(2–31)	65	1.50	(1–4)
*Dactylogyrus extensus*	65	1.25	(1–5)	70	3.00	(1–10)	55	0.95	(1–4)
*Dactylogyrus molnari*				70	5.20	(2–24)			
*Eudiplozoon nipponicum*	5	0.05	(1)	95	3.25	(1–7)	5	0.05	(1)
Trematoda									
*Diplostomum pseudospathaceum*	5	0.05	(1)	5	0.05	(1)			
Cestoda									
*Atractolytocestus huronensis*	5	2.55	(51)	20	1.95	(1–25)	5	0.10	(2)
*Khawia sinensis*							5	0.10	(2)
*Valipora campylancristrota*	5	0.05	(1)	15	0.15	(1)			
Crustacea									
*Argulus coregoni*				60	0.7	(1–3)			
*Argulus foliaceus*				45	0.6	(1–3)			
*Argulus japonicus*				10	0.1	(1)			
*Ergasilus sieboldi*	5	0.05	(1)	90	9.45	(1–29)	5	0.05	(1)
Bivalvia									
*Anodonta* sp.							5	0.05	(1)
Hirudinea									
*Piscicola geometra*				5	0.05	(1)			

**Table 2:** Parasite community diversity indices for common carp from the T-T (persistent at the treatment site), C-C (persistent at the control site), and C-T (restocked from the control to treatment site for six months) fish groups.

	T-T	C-T	C-C
Parasite species richness	10	10	16
Overall parasite abundance	4302	7600	1439
Dominance index	0.933	0.974	0.277
Shannon–Wiener index	0.202	0.090	1.721
Equitability	0.088	0.039	0.621

**Table 3:** Results of PERMANOVA comparisons between T-T (persistent at treatment site), C-C (persistent at control site), and C-T (restocked from control to treatment site for six months) fish groups, based on similarities in parasite taxa. Df = degrees of freedom, SS = sums of squares, MS = means of squares, F = F-statistics, R^2^ = percentage of variability explained, *p* = *p*-value. Statistically significant *p*-values are marked in bold.

	Df	SS	MS	F	R^2^	*p*
C-T vs. T-T	1	0.089	0.089	2.92	0.071	0.058
Residuals	38	1.160	0.031		0.929	
Total	39	1.249			1	
						
C-C vs. C-T	1	3.855	3.856	172.57	0.820	**0.001**
Residuals	38	0.849	0.022		0.180	
Total	39	4.704			1	
						
C-C vs. T-T	1	3.740	3.740	156.35	0.804	**0.001**
Residuals	38	0.909	0.024		0.196	
Total	39	4.648			1	

**Table 4:** Fish biometric parameters, including standard length (SL), eviscerated body weight (We), condition factor (K), and hepatosomatic (HSI), splenosomatic (SSI) and gonadosomatic (GSI) indices, presented as mean (±SD) and range values for the T-T (persistent at Cezarka treatment pond), C-C (persistent at control site), and C-T (restocked from control to treatment site for six months) fish groups. *n* = number of fish.

	T-T			C-C			C-T		
Parameter	Mean ± S	*n*	Range	Mean ± SD	*n*	Range	Mean ± SD	*n*	Range
SL (mm)	306 ± 12	20	(287–328)	271 ± 17	20	(235–310)	305 ± 15	20	(245–359)
We (g)	663 ± 90	20	(530–845)	528 ± 116	20	(285–820)	646 ± 82	20	(323–820)
K	2.31 ± 0.15	20	(2.1–2.6)	2.62 ± 0.14	20	(2.2–2.8)	2.27 ± 0.18	20	(1.9–2.6)
HSI	2.56 ± 0.29	20	(2.2–3.2)	3.39 ± 0.58	20	(2.5–4.9)	2.62 ± 0.52	20	(1.3–3.7)
SSI	2.87 ± 0.82	20	(1.7–4.7)	3.85 ± 0.88	20	(2.4–5.3)	2.77 ± 0.76	20	(1.5–4.7)
GSI (m)	4.38 ± 1.88	9	(1.8–6.0)	1.87 ± 1.14	9	(0.3–4.5)	2.75 ± 1.31	10	(0.6–4.5)
GSI (f)	1.22 ± 1.84	11	(0.1–2.7)	0.73 ± 1.28	10	(0.1–1.4)	1.00 ± 1.32	9	(0.3–1.8)

**Table 5:** Results of PERMANOVA comparisons between T-T (persistent at treatment site), C-C (persistent at control site), and C-T (restocked from control to treatment site for six months) fish groups, based on similarities in the concentration of pharmaceutical compounds. Statistically significant *p*-values are marked in bold.

	Df	SS	MS	F	R^2^	*p*
C-T vs. T-T	1	12.190	12.187	1.485	0.038	0.179
Residuals	38	311.940	8.209		0.962	
Total	39	324.140			1	
						
C-C vs. C-T	1	59.663	59.663	10.228	0.211	**0.001**
Residuals	38	221.671	5.833		0.788	
Total	39	281.334			1	
						
C-C vs. T-T	1	44.089	44.089	12.158	0.242	**0.001**
Residuals	38	137.799	3.626		0.758	
Total	39	181.888			1	

**Table 6:** Correlation coefficients (RV) and *p*-values of their Monte Carlo permutation tests for co-inertia analyses COIA conducted separately for three fish groups. Significant associations are shown in bold.

	Parasites vs. Pharmaceuticals		Parasites vs. Biometry		Pharmaceuticals vs. Biometry	
Group	RV	*p*	RV	*p*	RV	*p*
T-T	**0.334**	**0.037**	0.151	0.597	0.112	0.741
C-T	**0.380**	**0.036**	0.185	0.526	0.196	0.560
C-C	0.278	0.135	0.204	0.317	0.110	0.711

## Data Availability

The data that support the findings of this study are available upon a reasonable request from the corresponding author (M.O.).

## Supplementary Materials

The following supporting information can be downloaded at: https://www.mdpi.com/article/10.3390/ani13091464/s1, Table S1. List of pharmaceutical compounds and their metabolites determined in common carp liver and brain tissue; Table S2. List of pharmaceutical compounds assessed in common carp but not detected in tissues.
