# Supplementary material for: Response of Parasite Community Composition to Aquatic Pollution in Common Carp (Cyprinus carpio L.): A Semi-Experimental Study
Source: Animals (Basel). 2023 Apr 25;13(9):1464. doi: 10.3390/ani13091464 (PMC10177495; doi:10.3390/ani13091464)
Supplement: Supplementary file 1 [file animals-13-01464-s001.zip › animals-2276289-supplementary.pdf]

animals

MDPI

Supplementary material

Table S1. List of pharmaceutical compounds and their metabolites determined in common carp liver and brain tissue from groups T-T (persistent at Cezarka treatment pond), C-C (persistent at control site) and C-T (restocked from control to treatment site for six months; n=20 for each group). Concentration values presented as mean (± SD) and range (min-max), along with number of fish with positive detection of a particular compound (N positive) and LOQ range in ng/g wet weight. LOQ = limit of quantification. N.M. not measured.

		Liver				Brain			
		**C-C group**	**T-T group**	**C-T group**	LOQ range	**C-C group**	**T-T group**	**C-T group**	LOQ range
**Antiepileptics**									
Carbamazepine	Mean ± SD	<LOQ	<LOQ	<LOQ		**0.14**	<LOQ	**0.13**	
	Range				*0.053-0.12*	0.14		0.13	*0.074-0.097*
	N positive					1		1	
**Antidepressants**									
Citalopram	Mean ± SD	<LOQ	**0.13±0.05**	**0.12±0.08**		<LOQ	**0.064±0.029**	**0.046±0.019**	
	Range		0.056-0.22	0.048-0.25	*0.034-1.2*		0.031-0.11	0.029-0.081	*0.018-0.024*
	N positive		11	7			11	8	
N-Desmethylcitalopram	Mean ± SD	**0.12 ±0.11**	**1.7±1.1**	**1.8±2.0**		<LOQ	**0.071±0.024**	**0.062±0.026**	
	Range	0.037-0.46	0.43-5.1	0.57-10	*0.022-0.75*		0.037-0.11	0.038-0.11	*0.026-0.035*
	N positive	13	19	20			8	5	
Clomipramine	Mean ± SD	<LOQ	**0.21±0.06**	**0.24±0.09**		<LOQ	**0.035±0.018**	**0.025±0.012**	
	Range		0.15-0.32	0.12-0.41	*0.085-0.19*		0.013-0.064	0.013-0.051	*0.0051-0.0067*
	N positive		7	7			12	10	
Mirtazapine	Mean ± SD	<LOQ	<LOQ	<LOQ		0.21	<LOQ	<LOQ	
	Range				*0.11-0.26*	0.21			*0.054-0.078*
	N positive					1			
Sertraline	Mean ± SD	<LOQ	**0.67±0.40**	**0.77±0.55**		**0.11±0.10**	**0.55±0.33**	**0.55±0.23**	
	Range		(0.95-1.6)	0.080-12	*0.055-0.14*	0.072-0.41	0.12-1.5	0.11-0.91	*0.063-0.082*
	N positive		17	16		11	18	16	
Trazodone	Mean ± SD	**1.5±2.2**	<LOQ	**0.11±0.05**		N.M.	N.M.	<LOQ	

	Range	0.076-9.9		(0.049-0.19)	*0.033-0.16*					*N.M.*
	N positive	19		9						
Venlafaxine	Mean ± SD	**0.057±0.029**	**0.66±0.33**	**0.51±0.28**			**0.031±0.010**	**0.18±0.08**	**0.13±0.06**	
	Range	0.017-0.11	0.18-1.4	0.11-0.92	*0.0094-0.039*		0.018-0.042	0.056-0.32	(0.041-0.25)	*0.065-0.085*
	N positive	15	20	20			12	20	19	
Norsertraline	Mean ± SD	<LOQ	<LOQ	<LOQ			<LOQ	**6.2±1.2**	**6.7±1.6**	
	Range				*1.9-4.2*			4.3-8.8	4.9-9.4	*3.6-4.7*
	N positive							10	12	
**Beta-blockers**										
Metoprolol	Mean ± SD	<LOQ	**0.52**	<LOQ			<LOQ	<LOQ	<LOQ	
	Range		0.52		*0.072-0.16*					*0.56-0.84*
	N positive		1							
Metoprolol acid	Mean ± SD	<LOQ	**0.98±0.32**	<LOQ			<LOQ	<LOQ	<LOQ	
	Range		0.60-1.5		*0.58-2.1*					*0.35-0.54*
	N positive		5							
Telmisartan	Mean ± SD	<LOQ	**1.2±0.3**	<LOQ			<LOQ	<LOQ	<LOQ	
	Range		0.84-1.5		*0.34-0.75*					*0.56-0.77*
	N positive		3							
**CNS stimulants**										
Caffeine	Mean ± SD	**14±30**	**4.9±1.4**	**2.9±0.5**			**3.3±0.9**	**19±16**	**3.4±1.1**	
	Range	2.7-120	3.4-7.1	2.4-3.3	*1.0-3.7*		2.4-4.5	2.7-35	2.3-4.5	*1.3-2.2*
	N positive	14	4	2			4	2	2	
**Anti-inflammatory drugs**										
Diclofenac	Mean ± SD	<LOQ	**1.3±0.8**	**1.2±0.3**			<LOQ	**0.43±0.02**	**0.39±0.02**	
	Range		0.61-3.0	0.90-1.6	*0.37-0.83*			0.39-0.45	0.37-0.43	*0.046-0.06*
	N positive		6	4				4	4	
**Analgesics**										
Tramadol	Mean ± SD	<LOQ	**0.27±0.14**	**0.26±0.11**			**0.12±0.01**	**0.19±0.10**	**0.13±0.03**	
	Range		0.11-0.62	0.16-0.45	*0.11-0.28*		0.11-0.12	0.10-0.40	0.10-0.17	*0.57-0.11*
	N positive		10	9			2	7	6	

Table S2. List of pharmaceutical compounds assessed in common carp but not detected in tissues, i.e. with concentrations below the LOQ.

**Antiepileptics**
10, 11 trans dihydro carbamazepine, 10, 11-dihydro carbamazepine, Carbamazepine 10, 11 epoxide, Oxcarbazepine
**Pharmaceutical drugs for enlarged prostate**
Alfuzosin
**Antidepressants**
Amitryptiline, Maprotiline, Mianserin, O-Desmethylvenlafaxine
**Beta-blockers**
Atenolol, Bisoprolol, Cilazapril, Diltiazem, Irbesartan, Propranolol, Valsartan, Verapamil
**Cholesterol lowering medications**
Atorvastatin, Bezafibrate, Fenofibrate
**Antibiotics**
Clarithromycin, Clindamycin, Clindamycin sulfoxide, Erythromycin, N1-Acetylsulfamethoxazole, N4-acetylsulfamethoxazole, Sulfamethazine, Sulfamethizole, Sulfamethoxazole, Trimethoprim
**Anti-Parkinson's drugs**
Biperiden, Ropinirole
**Antihistamines**
Cetirizine, Clemastine, Diphenhydramine, Fexofenadine, Orphenadrine
**Anxiolytics**
Clonazepam, Oxazepam
**Anticholinergics**
Dicycloverine
**Antiarrhythmics**
Disopyramide, Sotalol
**Hypoglycemics**
Glibenclamide, Glimepiride
**Antipsychotics**
Haloperidol
**Diarrhea medication**

Loperamide
**Alzheimer's drugs**
Memantine
**Antimycotic medication**
Miconazole
**Influenza antiviral medications**
Oseltamivir carboxylate
**Antimigraine drugs**
Pizotifen
**Antifungal medication**
Terbinafine
**Bronchodilatans**
Terbutaline
**Diuretics**
Triamterene
**Anti-dementia drugs**
Donepezil
